# Expression of semaphorin 3A and neuropilin 1 with clinicopathological features and survival in human tongue cancer

**DOI:** 10.4317/medoral.18168

**Published:** 2012-08-28

**Authors:** Xiao Song, Wei Zhang, Yang Zhang, Hong Zhang, Zhen Fu, Jin Ye, Lai Liu, Xiao Song, Yu Wu

**Affiliations:** 1 Ph.D, MDS Institute of Stomatology, Nanjing Medical University; 2 Ph.D, MDS Department of Oral and Maxillofacial Surgery, Stomatologic Hospital of Jiangsu Province; 3 Ph.D Department of Oral Special Consultation, Stomatologic Hospital of Jiangsu Province; 4 Ph.D, MDS, Ph.D. DDS. Department of Oral Pathology, Stomatologic Hospital of Jiangsu Province

## Abstract

Objective: To investigate the association between semaphorin 3A (SEMA 3A) and its receptor neuropilin 1 (NRP1) and the clinicopathologic characteristics of patients with tongue cancer. Study Design: Forty-three tongue squamous cell carcinoma specimens were included. Immunohistochemical staining of SEMA3A and NRP1 was performed on 15 normal tongue epithelium specimens and the 43 tumour specimens. Immunoreactivity was evaluated based on the staining intensity and distribution score. Statistical analyses were performed using Chi-squared and Spearman tests and Kaplan-Meier analysis. Results: SEMA3A was significantly down-regulated in tongue cancer compared with normal tongue (P=0.025), while NRP1 was over-expressed in tumours (P<0.001). SEMA3A expression inversely correlated with nodal metastasis (P=0.017). NRP1 expression did not correlate with any clinicopathological characteristics. Higher SEMA3A expression strongly predicted longer survival (P=0.005). Scores for the NRP1/SEMA3A ratio of ≥1 predicted shorter survival (P=0.045). Conclusions: Aberrant expression of SEMA3A and its receptor NRP1 might be involved in the development of tongue cancer and might be useful prognostic markers in this tumour type.

** Key words:**Semaphorin 3A, neuropilin 1, tongue, squamous cell carcinoma.

## Introduction

Tongue squamous cell carcinoma has been the leading type of oral cancer with the notorious features of early lymph node metastasis and poor survival. Although treatment, which includes surgery, chemotherapy and (or) radiotherapy, has been effective, long-term survival has not substantially improved (1-4).

Recently, a growing emphasis has been placed on the relationship between the nervous system and cancer because increasing evidence supports common genetic mechanisms involved in both cancer development and the progression of neurodegenerative disease (5). Interestingly, the nervous system might exert a potential influence on the development of cancer; environmental enrichment (EE) has been shown to significantly inhibit xenograft tumour growth, but the mechanism remains elusive (6).

Members of the semaphorin (SEMA) family, which were originally described as axon guidance molecules (7,8), have recently attracted increased attention by oncologists because of their roles in tumour growth and metastasis (9-13). SEMAs are secreted or membrane-bound proteins that can be classified into eight classes (sema1-sema7 and viral sema). Class 3 semaphorins (SEMA3) are the only secreted semaphorins in vertebrates. Through binding to their receptors, neuropilins (NRPs) and plexins, SEMAs may function as chemo-repellents or chemoattractants (7,8). In addition, other molecules can also interact with SEMAs or their receptors on the cell membrane, making it even more difficult to predict the exact function of a SEMA. For example, SEMA3B, SEMA3F, and SEMA4D have been shown to regulate tumour angiogenesis, growth and metastasisin different manners (14-16).

SEMA3A is considered to be a candidate tumour suppressor in some cancers. SEMA3A can inhibit the proliferation of malignant mesothelial cells, decrease the adhesion or migration of prostate or breast cancer cells, and promote apoptosis in leukemic T cells (17,18). However, the role of SEMA3A in tongue squamous cell carcinoma remains unclear. Therefore, we focused on the expression of SEMA3A and its receptor NRP1 in tongue cancer and the potential contribution of these molecules in the prediction of prognosis.

## Material and Methods

-Patients and tissue samples

Forty-three primary tongue squamous cell carcinoma biopsy specimens from patients diagnosed between 2000 and 2006 were obtained from the Department of Oral Pathology and the Department of Oral Maxillofacial Surgery in the Stomatologic Hospital of Jiangsu Province, Nanjing Medical University. None of the patients had received any form of tumour-specific therapy before surgery. The follow-up period ranged from 2 to 135 months with an average of 55.6 months and a median of 70 months. The end point in the analysis was carcinoma-related death. Among the 15 normal tongue mucosa, 5 were obtained from freshly injured tongue mucosa after trauma and 10 were obtained from the defect border after removal of a benign tongue tumour. All tissues were obtained with the consent of the patients. This study protocol was approved by the Ethics Committee (Institutional Review Board) of the Nanjing Medical University. We have read the Helsinki Declaration and have followed its guidelines in this investigation.

-Immunohistochemistry 

All specimens were fixed in 10% formaldehyde solution and embedded in paraffin. Each tissue section (4-5 um) from representative paraffin blocks was deparaffinised in xylene and rehydrated through an alcohol gradient. Endogenous peroxidases were blocked with 3% hydrogen peroxide. For antigen retrieval, the sections were processed by conventional microwave heating in 0.01 M sodium citrate retrieval buffer (pH 6.0) for 20 min. The sections were then incubated with a rabbit monoclonal Neuropilin primary antibody (1:100; Abcam, Cambridge, UK) or a rabbit polyclonal Semaphorin 3A primary antibody (1:100; Abcam, Cambridge, UK) overnight at 4°C and subsequently incubated with goat anti-rabbit second antibody (1:5000; Abcam, Cambridge, UK) for 30 min at room temperature. The sections were then washed three times with Phosphate-buffered Saline (PBS) (pH 7.2) for 3 min. The reaction product was developed with DAB and counterstained with haematoxylin. Immunoreactivity in the tissue was judged by the pathologist, who was blinded to the clinical data and other immunohistochemical results according to the revised criteria suggested by the World Health Organization.

-Evaluation of Immunoreactivity 

Immunoreactivity was semi-quantitatively evaluated using the staining intensity score and distribution score (19). The immunoreactive score was defined as the proportion score multiplied by the intensity score. The proportion score was defined as 0, negative; 1, <10%; 2, 11–50%; 3, 51–80%; or 4, >80% positive cells. The intensity score was defined as 0, negative; 1, weak; 2, moderate; or 3, strong. The total score ranged from 0 to 12. The immunoreactivity scores were divided into one of the following three groups based on the final score; negative immunoreactivity was defined as a total score of 0, low expression was defined as a total score of 1–4, and high expression was defined as a total score of >4.

-Statistical Analyses

Statistical analyses to compare two groups of data were performed using an unpaired Student’s t-test. Ratio analysis was per-formed with a chi-squared or Fisher’s exact test. The Spearman test was used in the correlation analysis. Overall survival was obtained using the Kaplan-Meier method and compared using the log-rank test. P-values less than 0.05 were considered statisti-cally significant.

## Results

-Expression of SEMA3A and NRP1 in tongue squamous cell carcinoma

The immunohistochemical staining results for SEMA3A and NRP1 are presented in  Table 1, Table 2.

**Table 1:**
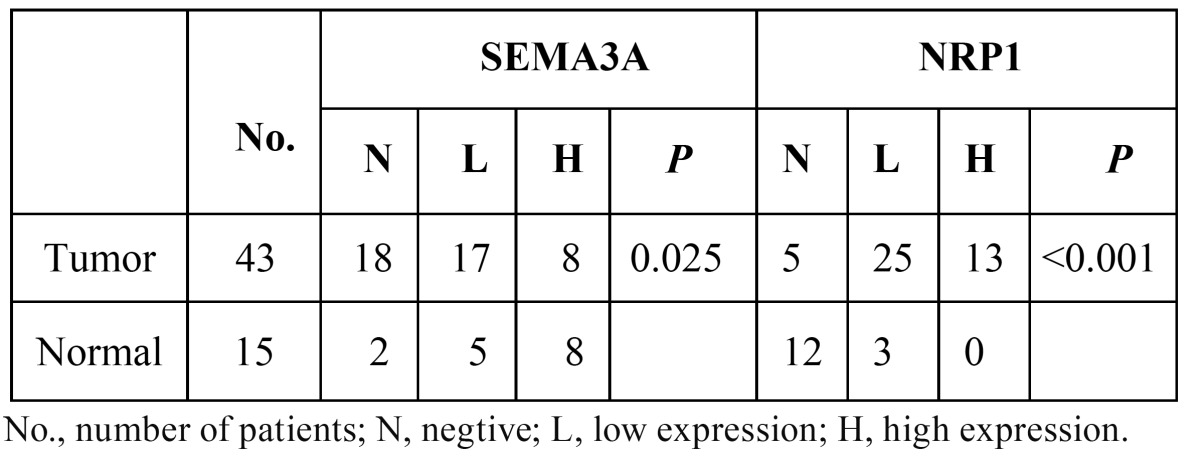
Expression of SEMA3A and NRP1 in normal tongue epithelum and tongue suqamous cell carcinoma.

**Table 2:**
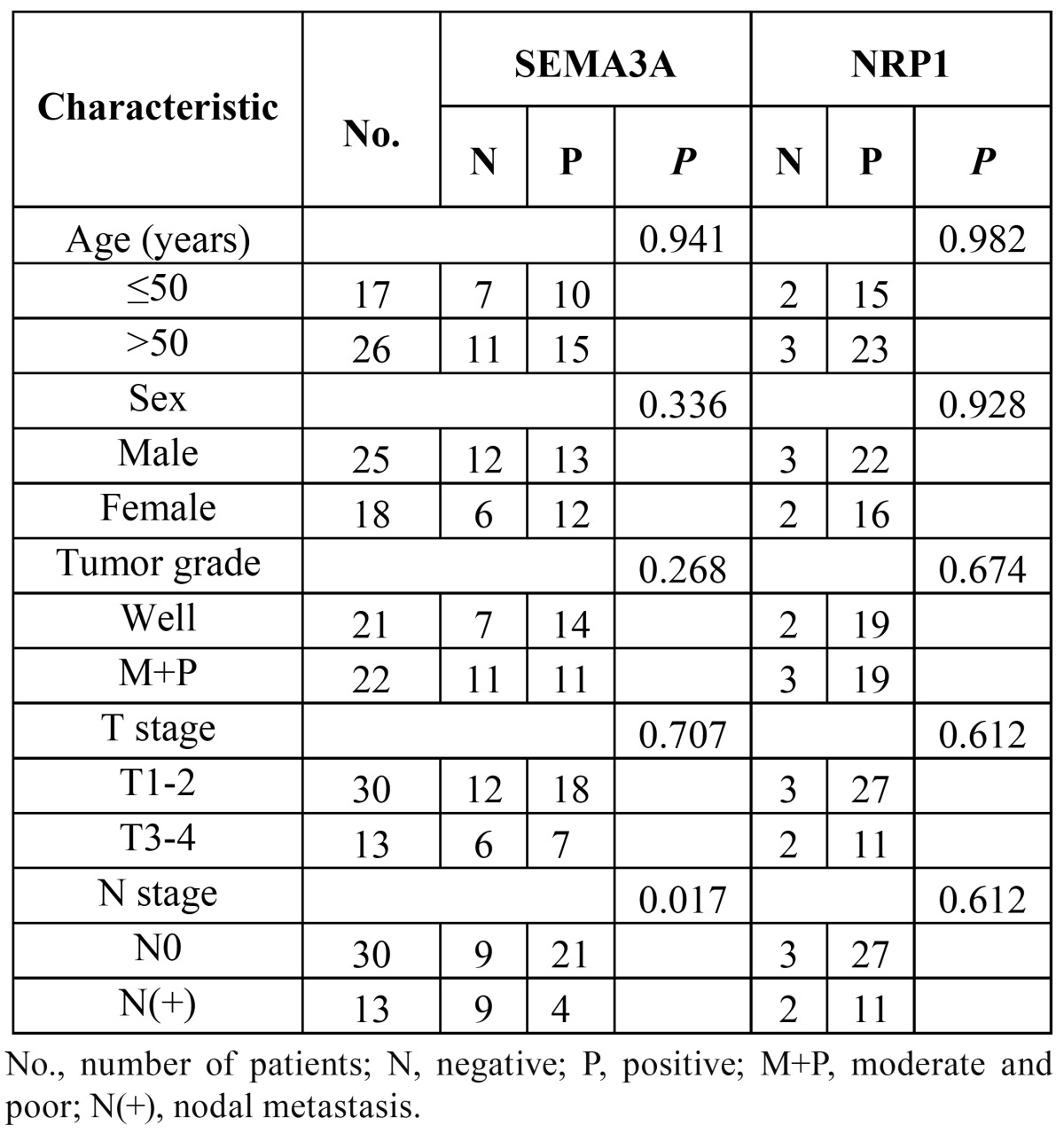
Relationship between SEMA3A and NRP1 expression levels of the tumors and clinical characteristic.

**Figure 1:**
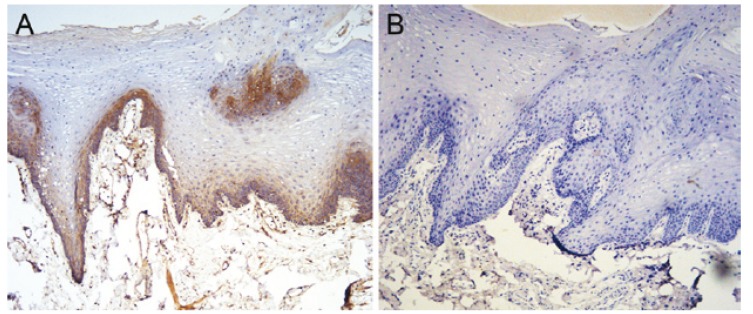
The expression of SEMA3A and NRP1 in normal tongue epithelium. SEMA3A was highly expressed in normal tongue epithelium, especially in the basal cell layer A) NRP1 was not expressed in normal epithelium B) (×400).

SEMA3A: SEMA3A was detected primarily in the nucleus and cytoplasm of the normal squamous tongue epithelium with mod-erate to strong immunoreactivity (Fig. 1), especially in basal membrane cells. Only 2 (13.3%) of the 15 normal tongue epithelium specimens showed negative staining ( Table 1). However, among the 43 tongue squamous cell carcinomas, negative staining was observed in 18 specimens (41.9%), high expression was observed in 8 (18.6%), and low expression was observed in 17 (39.5%). A significant difference in the expression of SEMA3A existed between normal and tumour tissues (P=0.025) ( Table 1).

**Figure 2:**
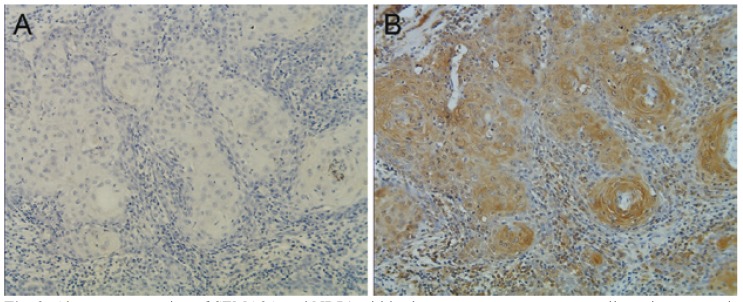
Aberrant expression of SEMA3A and NRP1 within the same tongue squamous cell carcinoma specimen. SEMA3A expression was negative A), while NRP1 was strongly immunostained B) (×400).

NRP1: Among the 15 normal tongue epithelium tissues, only 3 (20%) showed positive NRP1 expression (Fig. 1), while positive staining was observed in 38/43 (88.3%) tumour specimens. Among the tumour specimens, 25/43 (58.15%) displayed low expression and 13/43 (30.2%) displayed high expression. The NRP1 expression, which was observed in the membrane and cytoplasm, was located primarily in cancer nests. (Fig. 2) shows strongly positive NRP1 expression (Fig. 2) and negative SEMA3A expression (Fig. 2) within the same specimen. A significant difference existed between the expression of NRP1 in normal and tumour tissues (P<0.001) ( Table 1).

-Association between the expression of SEMA3A and NRP1 and clinicopathologic features

The relationship between the expression of SEMA3A and NRP1 is shown in table 2. A significant difference in SEMA3A expression was observed between the N0 and N(+) groups (P=0.017). Significantly lower expression of SEMA3A was observed in the lymph node metastasis group. We did not observe any significant difference in SEMA3A between the groups classified by age, sex or tumour T stages. Similarly, no significant difference in NRP1 was observed between the groups ( Table 2).

-Association between the expression of SEMA3A and NRP1 and survival

**Figure 3:**
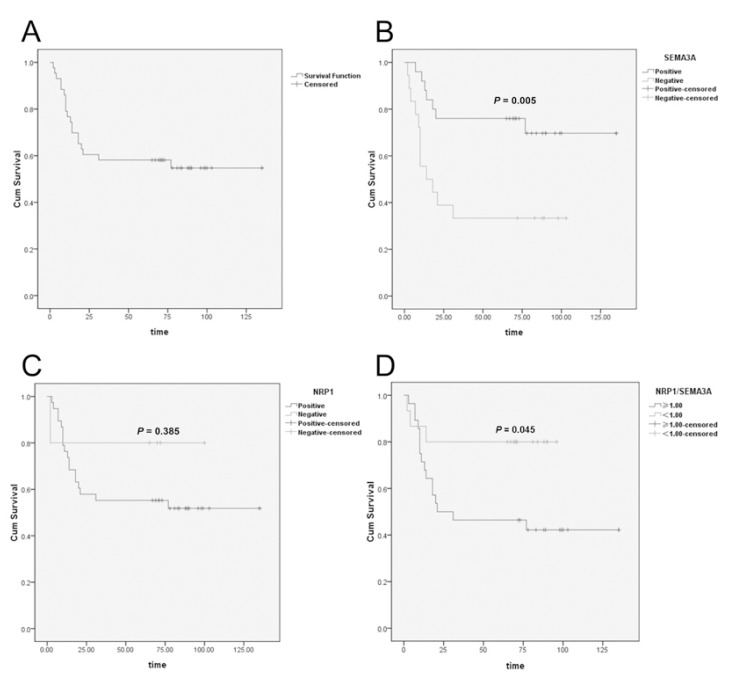
Analysis of SEMA3A and NRP1 expression and the survival of patients with tongue cancer. A) Overall survival curves of the 43 patients with tongue squamous cell carcinoma. B) Survival curves of the 43 patients with higher or lower expression of SEMA3A (log-rank test, P=0.005). C) Survival curves of the 43 patients with higher or lower expression of NRP1 (log-rank test, P=0.385). D) Survival curves of the 43 patients with NRP1/SEMA3A score ≥1 or <1 (log-rank test, P=0.045).

To the latest follow-up time, 23 of 43 (53.4%) patients were alive without recurrence, and 1 (2.3%) patient was alive with recur-rent disease. The other 19 patients had died from recurrent disease. The overall survival of the 43 patients is shown in figure 3. The 1-year and 5-year overall survival rates were 76.7% and 58.1%, respectively. High expression of SEMA3A predicted a significantly longer survival than low expression of SEMA3A (Fig. 3). However, the overall survival was not observed to be significantly different between the patients with high vs. low expression of NRP1 (Fig. 3). Interestingly, although no correlation existed between the expression of SEMA3A and NRP1 ( Table 3), in some patients, NRP1 was strongly positive while SEMA3A was almost totally absent (Fig. 2). Therefore, we compared the NRP1 and SEMA3A scores. Surprisingly, we found that patients with higher scores for the ratio of NRP1 to SEMA3A (NRP/SEMA3A ≥1) also showed a significantly shorter overall survival than those with scores <1 (Fig. 3).

**Table 3:**
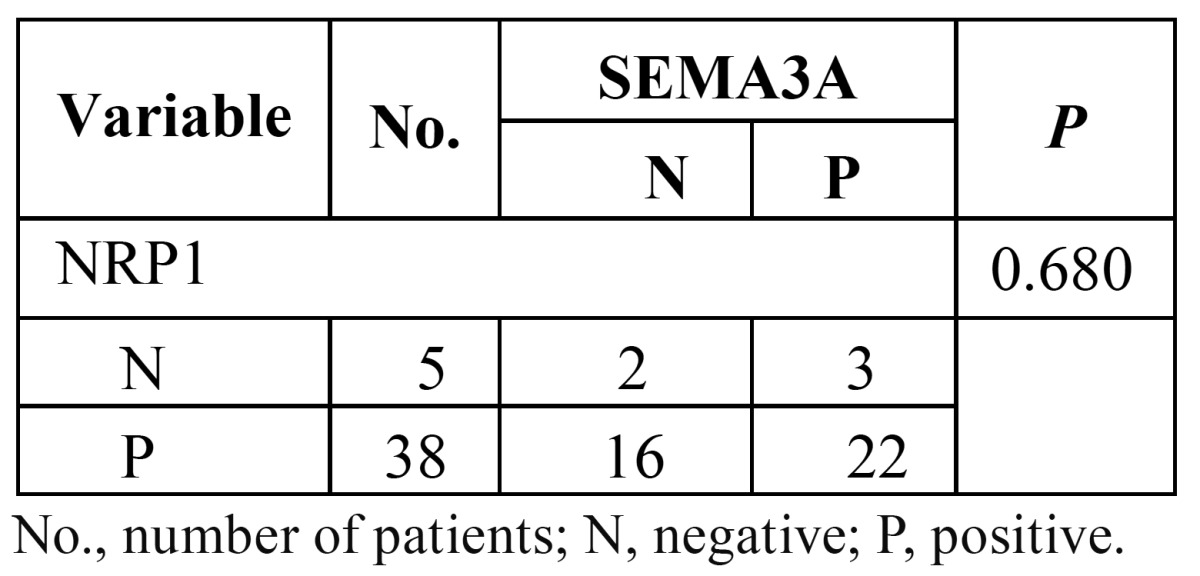
Correlation between expression of SEMA3A and NRP1 in tongue cancer.

## Discussion

Semaphorins and their receptors are involved in functions other than axonal guidance (20,21). However, the roles of SEMA3A and its receptor NRP1 have not been extensively studied, particularly in the long-term survival of patients with head and neck cancer. In this study, we focused on the most common type of oral cancer, tongue squamous cell carcinoma, and examined the expression of SEMA3A and NRP1. We found that no correlation existed between the expression of SEMA3A and NRP1 in tongue cancer specimens. However, loss of SEMA3A expression was observed in tongue cancer compared with normal tongue epithelium. The expression of the NRP1 receptor was remarkably elevated.

The discrepant change in SEMA3A signalling in tongue cancer in our study indicates the important role of this protein in the development of the disease. Moreover, lower expression of SEMA3A correlated with poor prognosis, suggesting a tumour suppressor function for SEMA3A in human tongue cancer. In fact, SEMA3A has been implicated as a tumour suppressor in other types of cancer (22-24). The mechanism involved in the tumour inhibition by SEMA3A might relate to its interaction with integrins. For example, in breast cancer, SEMA3A can inhibit cell attachment and cell migration by affecting the activation or stabilisation of surface integrins. Inhibition of integrins by SEMA3A could result in a blockade of endothelial and tumour cell migration, leading to reduced tumour angiogenesis and metastasis (25,26). In our study, we also found that SEMA3A expression inversely correlated with lymph node metastasis. This result indicates a role for SEMA3A in blocking tumour cell migration and metastasis. However, Pan et al. (22) have found that through binding to NRP1, SEMA3A can also suppress breast tumour cell migration by increasing the expression of integrin a2b1 in an autocrine fashion These conﬂicting results may reflect an ability of SEMA3A to differentially impact the adhesion of different cell types. In a more recent study on miRNA, Gaziel-Sovran A (27) have observed that miR-30b/30d upregulation correlates with higher metastatic potential, shorter time to recurrence, and reduced overall survival. Among the target genes of miR-30b/30d, the authors found a significant down-regulation of SEMA3A. Together with our results, these data indicate that SEMA3A might also be involved in modulating the epithelial to mesenchymal transition and metastasis.

The ability of SEMA3A to inhibit tumour angiogenesis by competing with vascular endothelium growth factor (VEGF) for binding with NRP1 has been more intensively studied and confirmed. Because VEGF is often up-regulated in a majority of malignancies, including head neck cancer, SEMA3A signalling might be inhibited by the binding of VEGF to NRP1. NRP1 is a single transmembrane glycoprotein with a molecular weight of approximately 130–140 kDa. NRP1 consists of a large extracellular domain, a short transmembrane domain and a short cytoplasmic domain. The binding domains for SEMA3A and VEGF are located in the extracellular domain and are named the A and B domains, respectively. However, SEMA3A can also bind to the B domain, which accounts for some of the observed functional competition between VEGF and SEMA3A for NRP1 binding (28). NRP1 has been reported to be up-regulated in many tumour types, and some clinical studies have shown that NRP1 overexpression is positively associated with metastatic potential, advanced stage, and clinical grade in prostate carcinoma, gastrointestinal carcinoma and colorectal carcinoma (29-32). In our study, NRP1 was poorly expressed in normal oral epithelium but was extensively up-regulated in tongue cancer tissue. However, this elevation did not correlate with any clinicopathologic characteristics, indicating its controversial role in tumour growth. The upregulation of NRP1 also did not correlate with significantly shorter survival. However, when combined with SEMA3A, we found that the ratio of NRP1 to SEMA3A also negatively correlated with survival. Specimens with higher NRP1 to SEMA3A expression scores corresponded to shorter survival. One explanation for this result may be that when NRP1 is expressed at higher levels than needed by SEMA3A, NRP1 predicts a worse prognosis. However, VEGF should also be taken into account because a balance between VEGF and SEMA3A is always present, and this balance modulates the cellular proliferation or apoptosis (33). If this balance is destroyed, SEMA3A often requires a higher concentration of NRP1 for binding than needed by VEGF (34). Therefore, we hypothesised that during the development of cancer, especially during the angiogenic process, the elevation of VEGF by tumour cells functions more than SEMA3A and NRP1 plays greater roles with VEGF than with SEMA3A. Therefore, VEGF and NRP1 expression increases and SEMA3A expression decreases. However, NRP1 is also involved in many other signalling pathways in addition to SEMA3A and VEGF. Therefore, much remains to be studied regarding the exact role of NRP1 and the relationship between the SEMA3s and VEGF in different types of cancer.

In conclusion, in this study, we observed the over-expression of NRP1 and loss of SEMA3A expression in human tongue cancer. Although the elevated expression of NRP1 did not correlate with any clinical characteristics in our study (more cases were potentially needed), lower expression of SEMA3A was strongly associated with worse patient survival. The combination analysis of SEMA3A together with NRP1 also provides a novel approach for assessing prognosis in this malignancy.
